# Knowledge structure and frontier trends of exercise interventions for Parkinson’s disease: a bibliometric and visualized analysis

**DOI:** 10.3389/fnagi.2026.1933587

**Published:** 2026-09-02

**Authors:** Jiangxi Yang, Huangyan Li, Shiliang Hu, Yang Xu, Yeting Zhang

**Affiliations:** 1 School of Aviation Sports, Civil Aviation Flight University of China, Guanghan, China; 2 School of Sports and Health, Guizhou Medical University, Guiyang, China; 3 School of Physical Education and Sports Science, Soochow University, Suzhou, China

**Keywords:** balance, gait, neurorehabilitation, non-motor symptoms, Parkinson’s disease, physical activity

## Abstract

**Background:**

Parkinson’s disease (PD) is a progressive neurodegenerative disorder with motor and non-motor manifestations. Exercise is increasingly integrated into the management of people with PD, yet the literature spans diverse intervention modalities, outcome domains, and mechanism-related topics.

**Objective:**

To characterize publication growth, global collaboration, article types, keyword networks, thematic evolution, outcome domains, and emerging research fronts in exercise intervention research for PD.

**Methods:**

English-language original articles and reviews indexed in the Web of Science Core Collection and Scopus from database inception to June 15, 2026, were retrieved. After eligibility screening, cross-database merging, and removal of duplicate and bibliographically incomplete records, 6,542 publications were included. Bibliometric analyses were conducted in R version 4.5.3 using the bibliometrix package and related R packages. The analyses included annual publication and citation trends, article-type distribution, geographic collaboration, keyword co-occurrence, thematic evolution, clinical and functional outcome mapping, citation structure, recent keyword bursts, and exploratory forecasting.

**Results:**

Publication output increased substantially after 2010, with original articles consistently comprising the majority of the literature and reviews increasing over time. Research output was concentrated in North America and Europe, while contributions from Asia expanded rapidly. Gait, rehabilitation, balance, motor function, and quality of life constituted the persistent thematic core. The gait–rehabilitation–balance family remained the largest thematic family in 2022–2026, accounting for 65.6% of publications, whereas the cognition–mood–quality-of-life family increased to 41.9%. Gait was the most frequently represented outcome, followed by balance, cognition, quality of life, motor function, and falls. Recent growth was observed in cognition, depression and anxiety, sleep, fatigue, evidence synthesis, technology-assisted rehabilitation, and exercise-dose optimization. Highly cited eligible publications primarily addressed exercise effectiveness, Tai Chi, cueing, treadmill and aerobic exercise, balance and resistance training, physical therapy, motor learning, and exercise-related neuroplasticity.

**Conclusion:**

Exercise research in PD has progressed from basic functional rehabilitation toward a broader framework integrating mobility, non-motor outcomes, technology-assisted delivery, and individualized exercise prescription. Future research should improve the reporting of intervention components and exercise dose, harmonize motor and non-motor outcome measurement, expand long-term and real-world follow-up, and evaluate mechanism- and subgroup-specific responses using rigorous clinical designs.

## 1 Introduction

Parkinson’s disease (PD) is a common progressive neurodegenerative disorder characterized pathologically by the progressive loss of dopaminergic neurons in the substantia nigra pars compacta and abnormal aggregation of α-synuclein (Kalia and Lang, 2015). Clinically, PD includes typical motor symptoms, such as bradykinesia, rigidity, resting tremor, and postural and gait disturbances, together with non-motor symptoms, including sleep disorders, depression, cognitive decline, autonomic dysfunction, and pain (Schapira et al., 2017). As the disease progresses, motor and non-motor symptoms interact and accumulate, impairing activities of daily living and quality of life among people with PD and increasing long-term care burden (Aamodt et al., 2024). Management goals have consequently shifted from isolated symptom control toward delaying functional decline, maintaining independence, and improving overall health status (Bloem et al., 2020).

The global prevalence and burden of PD have continued to increase (Li et al., 2025). Studies and projections based on Global Burden of Disease 2021 data indicate sustained growth in the number of people living with PD from 1990 to 2021, with approximately 25.2 million people projected to be living with the condition by 2050 (Luo et al., 2025; Su et al., 2025). Population aging, population growth, longer survival, improved diagnostic awareness, and disease registration may all contribute to this increase (Dorsey et al., 2018). Levodopa and other dopaminergic medications remain central to symptomatic treatment; however, their effects on gait impairment, postural instability, falls, reduced exercise tolerance, and some non-motor symptoms are limited (Connolly and Lang, 2014; Smulders et al., 2016). Motor complications, medication fluctuations, and axial symptoms can further compromise functional independence in the middle and advanced stages (Jankovic, 2005). Pharmacological treatment alone therefore cannot address all long-term functional needs of people with PD, supporting the development of safe, sustainable, and scalable non-pharmacological strategies.

Exercise, as an important component of rehabilitation therapy, has received increasing attention in recent years (Langeskov-Christensen et al., 2024). Unlike pharmacological treatment alone, exercise interventions may not only improve peripheral functional capacities, such as muscle strength, balance, gait, and cardiorespiratory fitness, but also influence disease-related dysfunction through central mechanisms, including neuroplasticity, brain network remodeling, and inflammatory regulation (Petzinger et al., 2013). Evidence from Cochrane systematic reviews and network meta-analyses suggests that most structured forms of exercise can improve motor symptoms and quality of life in people with PD; however, the relative advantages of different exercise modalities remain to be further clarified (Ernst et al., 2023).

Although the value of exercise interventions in the management of PD has been increasingly recognized, existing studies remain relatively fragmented with respect to intervention modalities, exercise dosage, outcome measures, and mechanistic explanations. Current research on exercise interventions for PD encompasses a wide range of modalities, including aerobic training, resistance training, balance training, dance, Tai Chi, and multimodal exercise, resulting in considerable heterogeneity in intervention design, outcome selection, and mechanistic focus. Previous studies have used bibliometric methods to examine the knowledge structure and hotspot trends of exercise-related research in PD, while others have summarized the publication characteristics of evidence on exercise interventions for people with PD from the perspective of systematic reviews (Rodrigues-Junior et al., 2023). Building on this foundation, the present study further focuses on the field of exercise interventions for PD and incorporates the most recent literature to visualize publication trends, country and institutional collaboration, core authors, keyword co-occurrence, and thematic evolution. In addition, this study summarizes research progress from the perspectives of exercise modality, major outcome measures, and potential mechanistic clues. By doing so, it aims to more specifically reveal research hotspots, evidence distribution, and future directions in this field, thereby providing references for optimizing exercise prescriptions, improving outcome assessment systems, and conducting mechanism-oriented clinical research.

## 2 Materials and methods

### 2.1 Search strategy

A comprehensive literature search was conducted in the Web of Science Core Collection (WoSCC) and Scopus on June 15, 2026. The search period extended from database inception to the search date. WoSCC and Scopus were selected because they provide broad multidisciplinary coverage and standardized bibliographic and cited-reference metadata, which are essential for bibliometric analysis and scientific knowledge mapping. The combined use of the two databases also reduced dependence on a single indexing source while maintaining sufficient comparability of bibliographic metadata. Only original articles and reviews published in English were considered eligible.

In WoSCC, the search was performed using the following topic-search strategy: TS = [(“Parkinson disease” OR “Parkinson’s disease” OR “idiopathic Parkinson disease” OR “idiopathic Parkinson’s disease”) AND (exercis* OR “physical activit*” OR “aerobic training” OR “resistance training” OR “strength training” OR “balance training” OR “gait training” OR treadmill* OR bicycl* OR cycling OR ergomet* OR dance OR dancing OR “tai chi” OR taiji OR qigong OR yoga OR boxing OR exergam* OR “virtual reality” OR telerehab* OR “tele-rehab*” OR “remote rehabilitation”)].

The initial WoSCC search identified 8,129 records. After excluding 1,214 records that were not classified as articles or reviews, 6,915 records remained. A further 726 non-English-language records were excluded, leaving 6,189 records. After excluding 1,961 ineligible publication materials or records with insufficient bibliographic information, 4,228 WoSCC records were retained.

In Scopus, the search was conducted in the title, abstract, and keyword fields using the following strategy: TITLE-ABS-KEY[(“Parkinson disease” OR “Parkinson’s disease” OR “idiopathic Parkinson disease” OR “idiopathic Parkinson’s disease”) AND (exercis* OR “physical activit*” OR “aerobic training” OR “resistance training” OR “strength training” OR “balance training” OR “gait training” OR treadmill* OR bicycl* OR cycling OR ergomet* OR dance OR dancing OR “tai chi” OR taiji OR qigong OR yoga OR boxing OR exergam* OR “virtual reality” OR telerehab* OR “tele-rehab*” OR “remote rehabilitation”)].

The initial Scopus search identified 6,628 records. After excluding 1,441 records that were not classified as articles or reviews, 5,187 records remained. A further 1,321 non-English-language records were excluded, leaving 3,866 records. After excluding 856 ineligible publication materials or records with insufficient bibliographic information, 3,010 Scopus records were retained.

**FIGURE 1:**
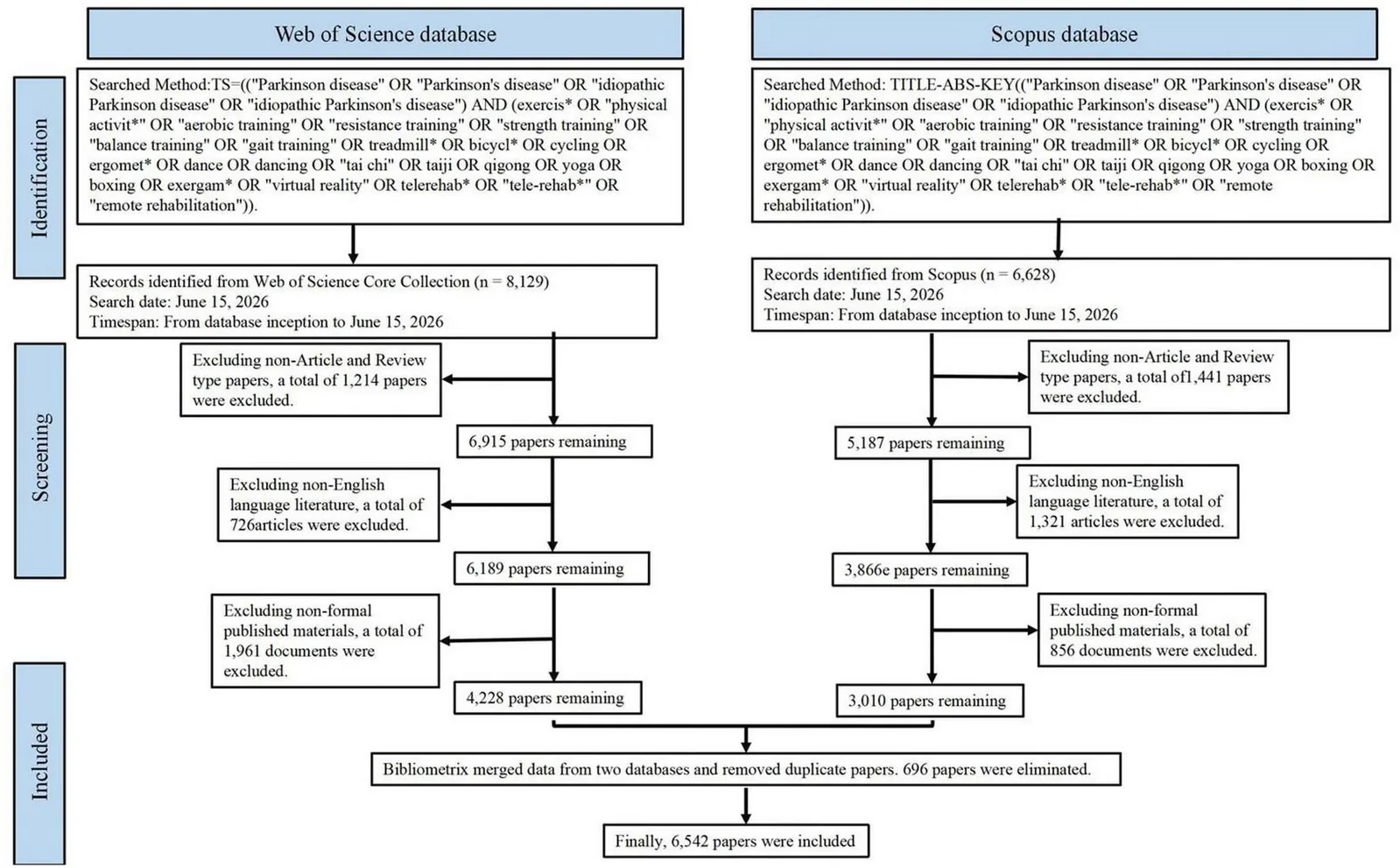
Flowchart of literature identification, cross-database processing, and bibliographic cleaning.

The 4,228 records from WoSCC and 3,010 records from Scopus were imported into R and merged using the bibliometrix package, resulting in 7,238 records before cross-database deduplication. Duplicate records were identified primarily by digital object identifiers. For records without a DOI, normalized titles, author names, and publication years were compared. After removing 696 duplicate records, 6,542 publications were included in the final bibliometric analysis. The literature identification and screening process is presented in Figure 1.

### 2.2 Inclusion and exclusion criteria

The inclusion criteria were: (1) original articles or reviews indexed in the Web of Science Core Collection or Scopus; (2) studies involving people with PD; (3) studies examining exercise, physical activity, physical training, exercise-based rehabilitation, or a related modality; and (4) English-language publications. Original research included randomized and non-randomized intervention studies and observational studies; review publications included systematic reviews, meta-analyses, and narrative reviews. No clinical outcome category was used as an independent eligibility criterion because the purpose was to map the full spectrum of outcomes investigated in exercise-related PD research. Outcome terms were classified after study inclusion.

The exclusion criteria were: (1) duplicate records; (2) publications clearly unrelated to exercise or physical activity in PD; (3) animal, cellular, or other preclinical studies without direct relevance to people with PD; (4) studies focused solely on pharmacotherapy, deep brain stimulation, or another non-exercise treatment; (5) studies of other neurological conditions without separately identifiable PD data; (6) conference abstracts, news reports, letters, editorials, book reviews, protocols without results, and other non-research publications; (7) records with insufficient bibliographic information for reliable analysis; and (8) withdrawn publications. Retracted publications were retained only when required to describe the retrieved citation record, were clearly labeled, and were excluded from substantive interpretation.

### 2.3 Data processing and bibliometric analysis

Bibliographic records were imported into R 4.5.3. The bibliometrix package was used for format conversion, merging, and deduplication, and correspondence between original and cleaned fields was retained for traceability. Author and institutional names were standardized where variants could be reliably identified. Article type was classified as original research or review using database document-type fields and was analyzed over time.

Keyword data were drawn from author keywords, Keywords Plus, and Scopus indexed keywords. Terms were converted to lowercase; punctuation, redundant spaces, singular/plural variants, abbreviations, hyphenated expressions, and synonyms were standardized using a predefined replacement thesaurus. Core search terms and generic indexing terms, including PD, exercise, physical activity, human, humans, article, review, male, and female, were removed before hotspot analyses. Demographic descriptors such as aged and middle aged were retained because age distribution was part of the descriptive mapping. Terms representing independent conditions, such as Alzheimer disease and stroke, were not classified as PD outcomes and were excluded from thematic interpretation when unrelated to PD. Levodopa and dopamine terms were retained only as treatment-context descriptors within eligible exercise studies and were not interpreted as pharmacological intervention themes.

All bibliometric analyses were conducted in R version 4.5.3 using the bibliometrix package and related R packages. These analyses included annual publication and citation trends, article-type distribution, country and institutional collaboration, keyword frequency and co-occurrence, thematic clustering and evolution, outcome mapping, burst detection, and scientific knowledge mapping. Data processing and keyword standardization were performed using dplyr, tidyr, and stringr; network analyses were conducted using igraph; and visualizations were generated using ggplot2, ggraph, ggrepel, ggalluvial, and treemapify. Exploratory forecasts of keyword occurrence were generated using the forecast package. Four periods were prespecified according to inflection points in the annual publication trajectory and were applied consistently across the period-based analyses: 1983–2005 (emergence), 2006–2014 (expansion), 2015–2021 (rapid growth), and 2022–2026 (current phase). These cut points were selected to capture major changes in publication growth while retaining a sufficient number of records within each interval.

## 3 Results and analysis

### 3.1 Annual publication output and citation trends

**FIGURE 2:**
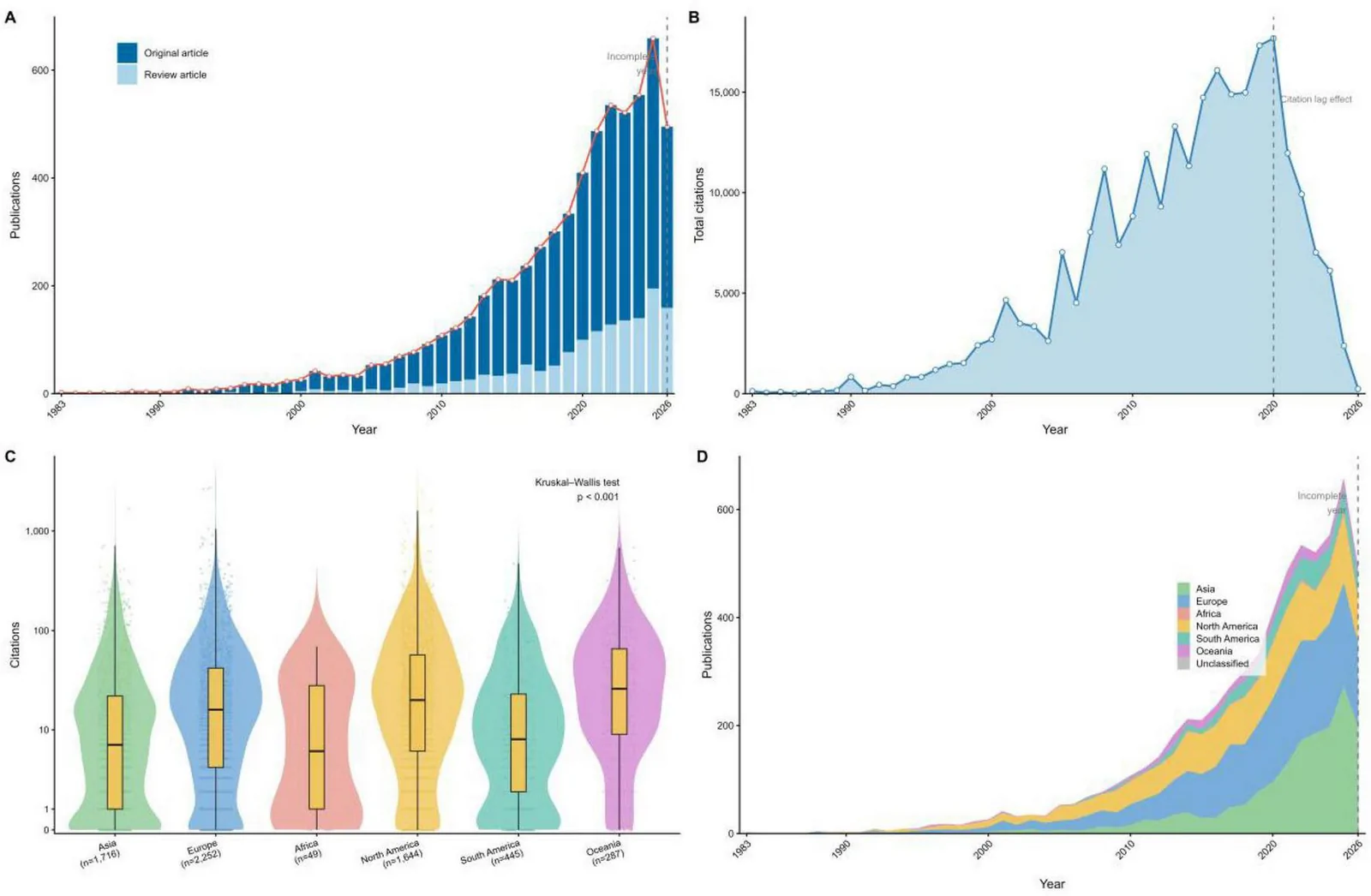
Annual publication output, citation trends, and geographic distribution of research on exercise and PD. **(A)** Annual numbers of original and review articles; stacked bars indicate publication type and the overlaid line indicates total output. **(B)** Total citations accumulated by publications according to publication year. **(C)** Citation distributions across continents; violin width indicates density, embedded boxplots indicate medians and interquartile ranges, and citation counts are displayed on a logarithmic scale. Differences were assessed using the Kruskal–Wallis test (*p* < 0.001). **(D)** Annual publication output by continent. “Unclassified” denotes records without sufficient geographic information. The dashed line marks the incomplete 2026 observation period.

Annual publication output increased substantially over the study period (Figure 2A). Output was low during the early years, rose gradually from the late 1990s, and accelerated after 2010. Original articles constituted the majority of publications in each year, while the number of reviews also increased over time. The lower count in 2026 reflects the June 15 search cutoff.

Total citations varied across publication years (Figure 2B). Publications issued from the early 2000s onward accumulated increasing citation totals, with the largest annual totals observed among papers published in the late 2010s. Publications from the most recent years had lower accumulated citation counts at the search date.

Citation distributions differed across continents (Figure 2C; Kruskal–Wallis test, *p* < 0.001). Each continental group displayed a right-skewed distribution, with most publications receiving modest citation counts and a smaller number forming the upper tail. Europe and North America had broader distributions than regions with fewer publications. These comparisons were based on unadjusted publication-level citation counts.

The geographic composition of annual publication output also changed over time (Figure 2D). Europe and North America contributed the largest shares during most of the observation period, while Asia accounted for a progressively larger share in recent years. Contributions from South America, Oceania, and Africa were smaller. Records lacking sufficient affiliation information were classified as unclassified, and the 2026 values were incomplete at the search date.

### 3.2 Global research output and international collaboration

**FIGURE 3:**
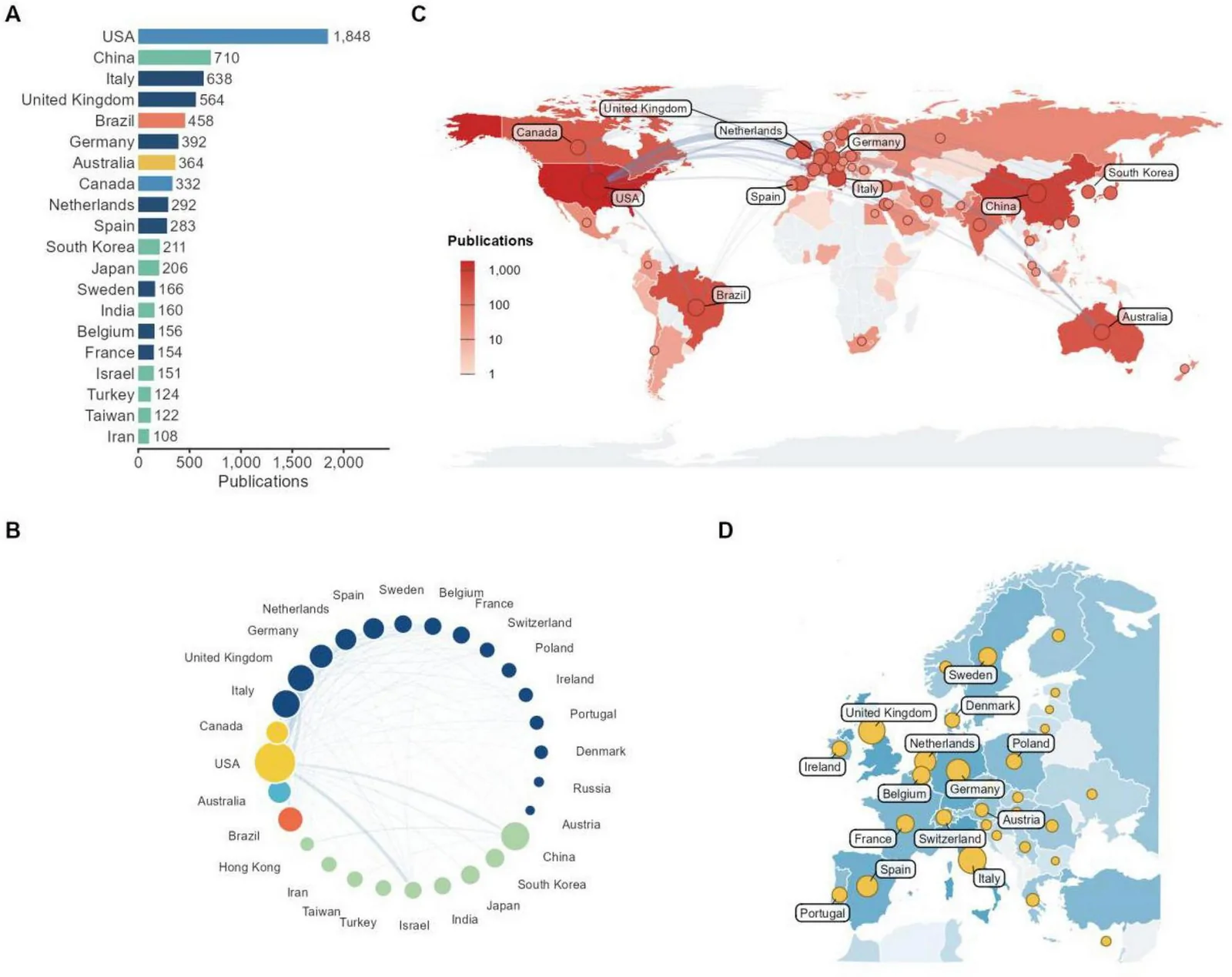
Geographic distribution and international collaboration in research on exercise and PD. **(A)** The 20 countries with the largest absolute publication counts. **(B)** Country-level collaboration network; node size represents publication output and line thickness represents collaboration strength. **(C)** Global publication distribution and cross-national collaboration; shading and circle size indicate publication output and curved lines indicate collaboration. **(D)** Enlarged European collaboration network. Country counts were not normalized by population size or researcher workforce.

Publication output was concentrated in a limited number of countries (Figure 3A). The United States ranked first with 1,848 publications, followed by China (*n* = 710), Italy (*n* = 638), the United Kingdom (*n* = 564), and Brazil (*n* = 458). Germany, Australia, Canada, the Netherlands, and Spain also contributed substantial output. These values are absolute publication counts and were not normalized by national population size or researcher workforce.

The international collaboration network contained extensive cross-country connections (Figure 3B). The United States formed the largest node and maintained links with countries across Europe, Asia, North America, and Oceania. The United Kingdom, Italy, Germany, the Netherlands, Spain, Sweden, Belgium, France, and Switzerland formed a dense European cluster. China was a major Asian hub with both regional and intercontinental links.

The global map located the largest publication concentrations in North America, Western Europe, East Asia, and Australia (Figure 3C). Dense transatlantic links connected North America and Europe, while additional collaboration extended to East Asia, South America, and Oceania. Publication output and collaboration were comparatively sparse across much of Africa and parts of Central Asia.

Within Europe, activity and collaboration were concentrated in Western and Central Europe (Figure 3D). The United Kingdom, the Netherlands, Belgium, Germany, France, Switzerland, Italy, Spain, Austria, Denmark, and Sweden formed the principal regional network, with Germany, the United Kingdom, the Netherlands, Belgium, France, Italy, and Switzerland occupying prominent positions.

### 3.3 Distribution of clinical and functional outcome themes

**FIGURE 4:**
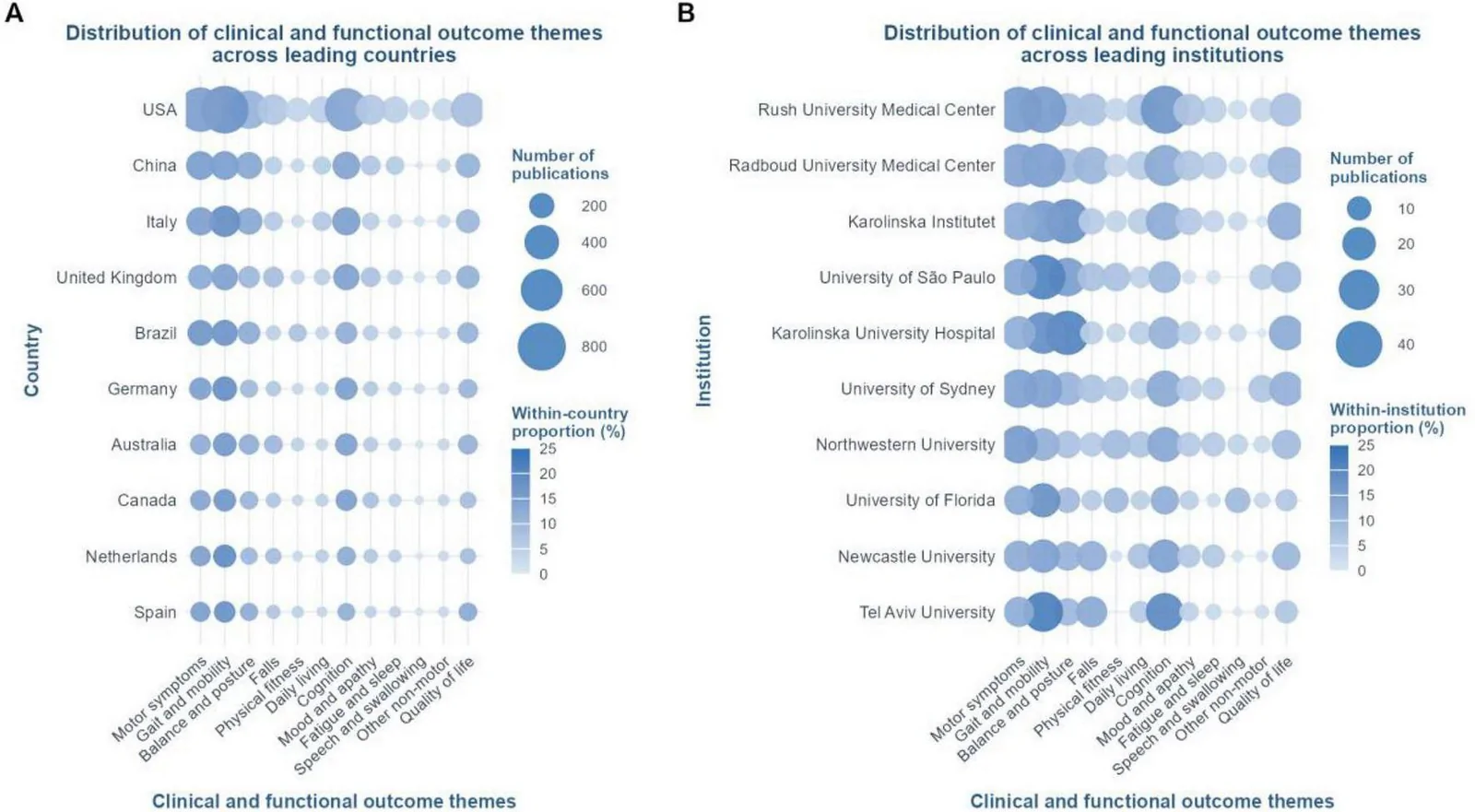
Distribution of clinical and functional outcome themes across leading countries and institutions in research on exercise and PD. **(A)** Outcome themes across the 10 leading countries. **(B)** Outcome themes across the 10 leading institutions. Bubble size represents the number of publications associated with each theme, and color intensity represents the within-country or within-institution proportion. Publications addressing multiple domains could be assigned to more than one theme, but each publication was counted once within each theme.

The distribution of clinical and functional outcome themes varied across the leading countries (Figure 4A). Motor symptoms, gait and mobility, cognition, and quality of life were the most frequently represented domains. The United States contributed the largest absolute number of publications in most categories, while China, Italy, the United Kingdom, and Brazil also covered multiple domains. Speech and swallowing, other non-motor symptoms, physical fitness, and falls generally accounted for smaller shares. Within-country proportions indicated differences in thematic emphasis beyond differences in total output.

A multidimensional but heterogeneous pattern was also evident among leading institutions (Figure 4B). Rush University Medical Center, Radboud University Medical Center, Karolinska Institutet, the University of São Paulo, and Karolinska University Hospital contributed across most outcome domains. Motor symptoms, gait and mobility, balance and postural control, cognition, and quality of life predominated. Speech and swallowing and other non-motor outcomes were less frequently represented.

### 3.4 Thematic structure and temporal evolution of keywords

**FIGURE 5:**
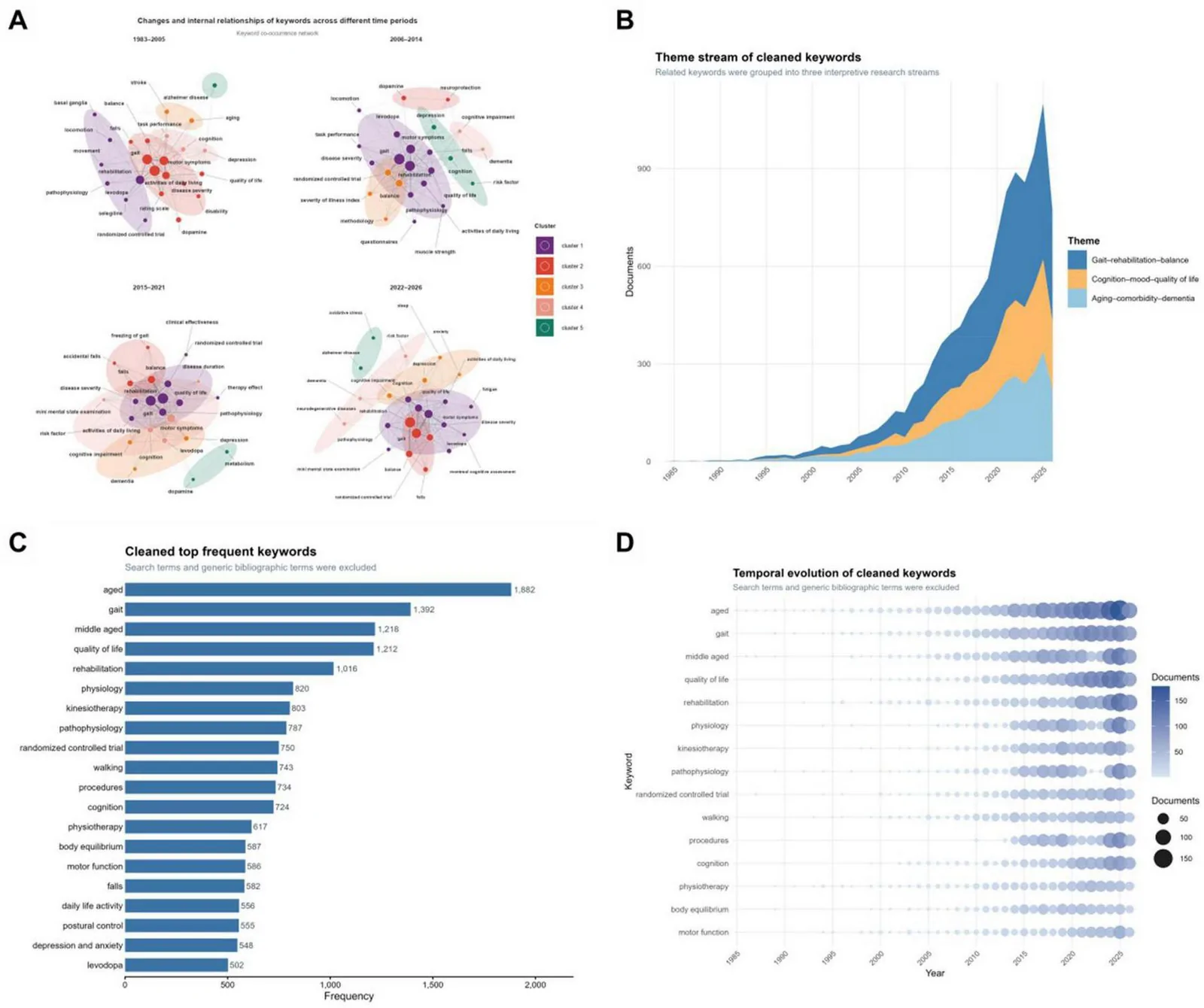
Temporal evolution and thematic patterns of keywords in exercise-related PD research. **(A)** Keyword co-occurrence networks across 1983–2005, 2006–2014, 2015–2021, and 2022–2026; node size indicates frequency, links indicate co-occurrence, and colors distinguish clusters. **(B)** Annual output of the gait–rehabilitation–balance, cognition–mood–quality of life, and aging–comorbidity–dementia streams. **(C)** The 20 most frequent cleaned keywords. **(D)** Annual distribution of high-frequency keywords; bubble size and color intensity indicate the number of documents.

After search terms and generic bibliographic terms were removed, the keyword networks expanded and became more interconnected across the four periods (Figure 5A). The 1983–2005 network was relatively sparse and centered on gait impairment, rehabilitation, clinical severity, and physiological or pathological characteristics; treatment-context terms also appeared. During 2006–2014, gait, walking, rehabilitation, motor symptoms, quality of life, depression, cognitive impairment, falls, and disease severity formed several connected clusters. In 2015–2021, rehabilitation and functional topics became more central and were linked with balance, falls, activities of daily living, cognition, and non-motor symptoms. In 2022–2026, gait, rehabilitation, balance, motor function, quality of life, cognition, and randomized controlled trials formed the main research core, while technology-assisted assessment and intervention terms appeared at the network periphery.

The cleaned keywords were grouped into three broad streams: gait–rehabilitation–balance, cognition–mood–quality of life, and aging–comorbidity–dementia (Figure 5B). All three streams increased after 2010. Gait–rehabilitation–balance remained the largest stream, while cognition–mood–quality of life expanded steadily. The 2026 values were based on a partial publication year.

The most frequent cleaned keyword was aged (*n* = 1,882), followed by gait (*n* = 1,392), middle aged (*n* = 1,218), quality of life (*n* = 1,212), and rehabilitation (*n* = 1,016) (Figure 5C). Other frequent terms included physiology, kinesitherapy, pathophysiology, randomized controlled trial, walking, procedures, cognition, physiotherapy, body equilibrium, motor function, falls, daily life activity, postural control, depression and anxiety, and levodopa.

Annual keyword trajectories indicated sustained increases in gait, quality of life, rehabilitation, walking, cognition, randomized controlled trial, physiology, kinesitherapy, pathophysiology, body equilibrium, and motor function, particularly after 2010 (Figure 5D). Age-related descriptors remained frequent across the observation period.

### 3.5 Growth trends, forecasts, and recent keyword bursts

**FIGURE 6:**
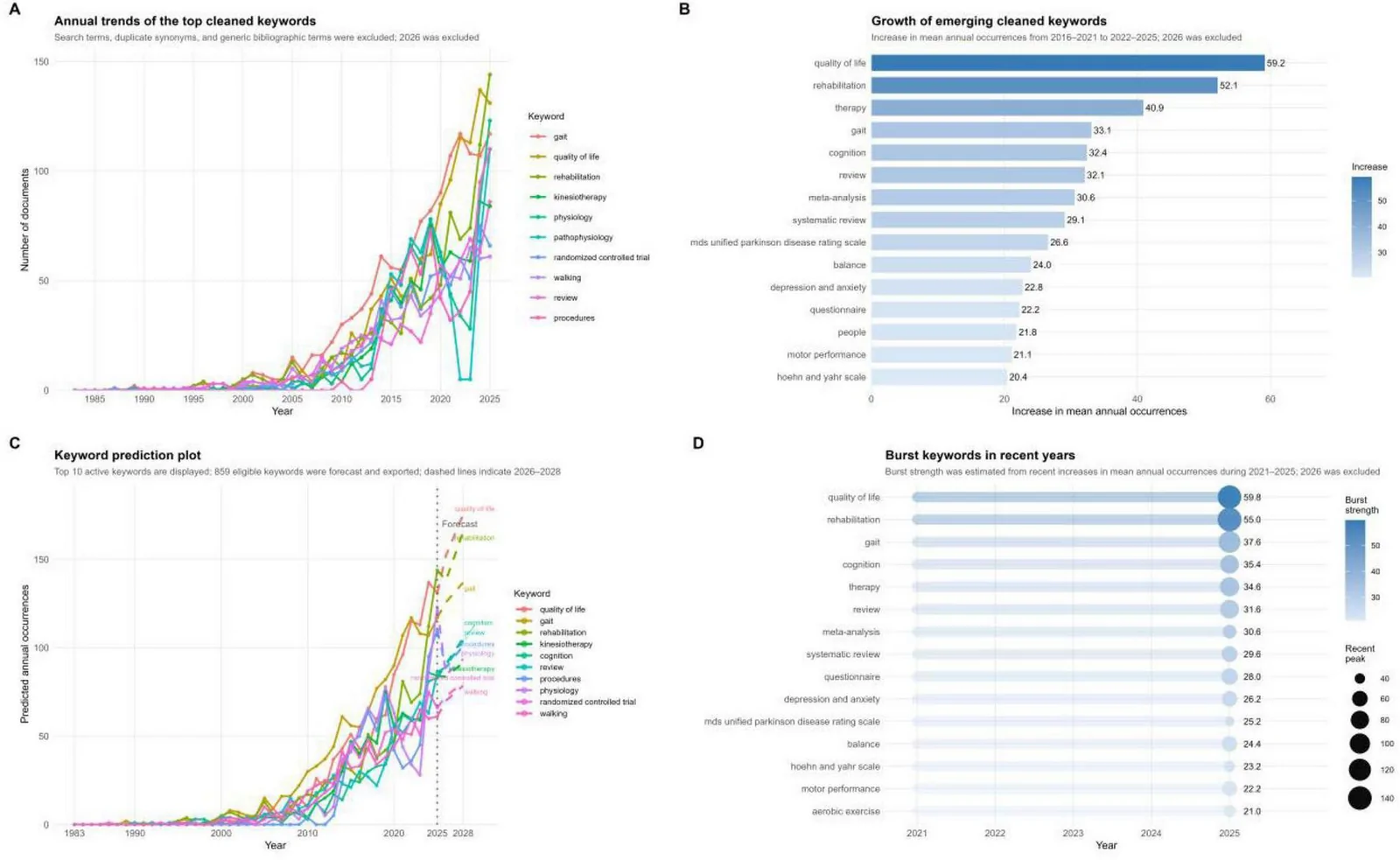
Annual trends, emerging growth, forecasting, and recent bursts of keywords in exercise-related PD research. **(A)** Annual occurrences of the 10 leading cleaned keywords. **(B)** Largest increases in mean annual occurrences between 2015–2021 and 2022–2025; bar length and color intensity indicate the magnitude of increase. **(C)** Forecasts for the 10 most active keywords selected from 859 eligible terms; solid lines represent observed values and dashed lines represent forecasts for 2026–2028. **(D)** Strongest bursts during 2021–2025; bubble size represents recent peak occurrence and color intensity represents burst strength. Search terms, duplicate synonyms, and generic bibliographic terms were excluded. Data from 2026 were excluded from the observed growth and burst calculations because indexing was incomplete.

Annual occurrences of the leading cleaned keywords were low before the mid-2000s, increased after 2010, and rose more rapidly after 2015 (Figure 6A). Quality of life, rehabilitation, gait, kinesitherapy, physiology, pathophysiology, walking, and randomized controlled trial all increased over the long term, with fluctuations in recent years.

Comparison of mean annual occurrences between 2015–2021 and 2022–2025 identified the largest increases for quality of life (+59.2), rehabilitation (+52.1), and therapy (+40.9) (Figure 6B). Gait (+33.1), cognition (+32.4), review (+32.1), meta-analysis (+30.8), and systematic review (+29.1) also increased.

Exploratory forecasting of 859 eligible cleaned keywords projected continued growth during 2026–2028 (Figure 6C). Quality of life, gait, and rehabilitation had the largest projected increases among the 10 displayed keywords. Kinesitherapy, cognition, review, physiotherapy, randomized controlled trial, procedures, and walking were also projected to remain prominent. These values are model-based estimates rather than observed publication counts.

Recent burst analysis identified the strongest 2021–2025 bursts for quality of life (59.8), rehabilitation (58.0), and gait (57.6), followed by cognition (36.4) and therapy (34.6) (Figure 6D). Review, meta-analysis, systematic review, depression and anxiety, balance, motor performance, clinical rating scales, and aerobic exercise also appeared among the recent burst terms.

### 3.6 Knowledge base, outcome evolution, and strategic thematic structure

**FIGURE 7:**
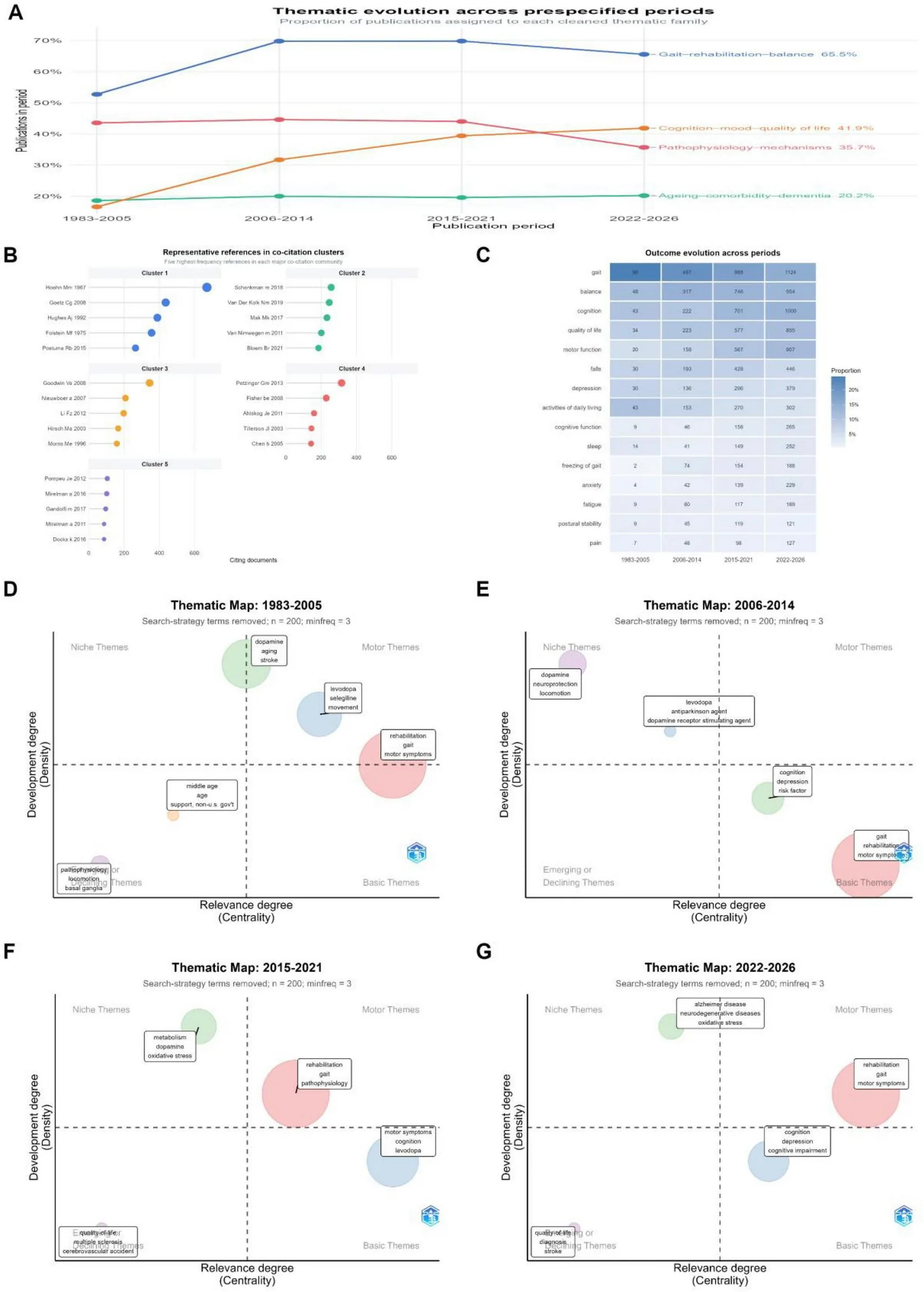
Thematic evolution, intellectual structure, and outcome development in exercise-related PD research. **(A)** Proportions of publications assigned to four predefined thematic families across 1983–2005, 2006–2014, 2015–2021, and 2022–2026; percentages are not mutually exclusive. **(B)** Representative highly cited references within five co-citation clusters; the horizontal axis indicates citing documents and colors distinguish clusters. **(C)** Evolution of major clinical and functional outcomes; cell values indicate publication counts and color intensity indicates the within-period proportion. **(D–G)** Strategic thematic maps for the four periods. The horizontal axis represents centrality and the vertical axis represents density. Quadrants indicate niche themes, motor themes, emerging or declining themes, and basic themes. Bubble size reflects keyword frequency and bubble color distinguishes clusters.

The relative prominence of the four predefined thematic families varied across the four publication periods (Figure 7A). The proportion of publications assigned to the gait–rehabilitation–balance family increased from approximately 52% in 1983–2005 to nearly 70% in both 2006–2014 and 2015–2021, before reaching 65.6% in 2022–2026. The cognition–mood–quality-of-life family increased steadily from approximately 16% in the earliest period to 41.9% in 2022–2026. The pathophysiology–mechanisms family remained relatively stable during the first three periods and accounted for 35.7% of publications in the most recent period. The aging–comorbidity–dementia family remained close to one-fifth of the literature and accounted for 20.2% of publications in 2022–2026. Because individual publications could be assigned to more than one thematic family, these percentages were not mutually exclusive.

Reference co-citation analysis identified five clusters within the intellectual structure of the field (Figure 7B). Cluster 1 contained the references cited by the largest number of retrieved publications, whereas the remaining clusters represented smaller but distinguishable bodies of clinical, rehabilitation, methodological, and mechanism-related literature.

The number of publications addressing both motor and non-motor outcomes increased across the four periods (Figure 7C). Gait was the most frequently investigated outcome, followed by balance, cognition, quality of life, motor function, and falls. Depression, activities of daily living, cognitive function, sleep, freezing of gait, anxiety, fatigue, postural stability, and pain were less frequently represented during the earliest period but occurred more often in later periods.

The strategic maps documented temporal changes in thematic centrality and density (Figures 7D–G). In 1983–2005, rehabilitation, gait, and motor symptoms were centrally positioned, whereas aging-related and pathophysiological clusters were more specialized or peripheral. During 2006–2014, gait, rehabilitation, and motor symptoms occupied the basic-theme quadrant, while cognition, depression, and risk-factor terms became increasingly relevant.

During 2015–2021, the rehabilitation–gait–pathophysiology cluster occupied the motor-theme quadrant, indicating both high centrality and strong internal development. A separate cluster containing motor symptoms, cognition, and levodopa was highly central but less internally developed. In this context, levodopa reflected medication status or standard-care conditions reported within eligible exercise studies rather than a separate pharmacological intervention category. During 2022–2026, rehabilitation, gait, and motor symptoms remained the principal motor theme, while cognition, depression, and cognitive impairment formed an increasingly central basic theme.

After relevance screening, 17 highly cited publications were retained as directly related to exercise, physical therapy, or exercise-based rehabilitation for people with Parkinson disease (Table 1). Their citation counts ranged from 319 to 741. The most highly cited publication was the systematic review and meta-analysis by Goodwin et al. on the effectiveness of exercise interventions for people with Parkinson disease, with 741 citations. This was followed by the randomized trial of Tai Chi for postural stability by Li et al. with 728 citations, the RESCUE trial of home-based cueing training by Nieuwboer et al. with 718 citations, and the review of exercise-enhanced neuroplasticity by Petzinger et al. with 675 citations.

**TABLE 1:** The most highly cited publications on exercise and exercise-based rehabilitation for people with Parkinson disease.

Rank	First author	Year	Article title	Journal	Citations
1	Goodwin V. A.	2008	The effectiveness of exercise interventions for people with Parkinson’s disease: a systematic review and meta-analysis	Movement Disorders	741
2	Li F.	2012	Tai chi and postural stability in patients with Parkinson’s disease	New England Journal of Medicine	728
3	Nieuwboer A.	2007	Cueing training in the home improves gait-related mobility in Parkinson’s disease: the RESCUE trial	Journal of Neurology, Neurosurgery and Psychiatry	718
4	Petzinger G. M.	2013	Exercise-enhanced neuroplasticity targeting motor and cognitive circuitry in Parkinson’s disease	The Lancet Neurology	675
5	Thaut M. H.	1996	Rhythmic auditory stimulation in gait training for Parkinson’s disease patients	Movement Disorders	578
6	van der Kolk N. M.	2019	Effectiveness of home-based and remotely supervised aerobic exercise in Parkinson’s disease: a double-blind, randomized controlled trial	The Lancet Neurology	463
7	Schenkman M.	2018	Effect of high-intensity treadmill exercise on motor symptoms in patients with *de novo* Parkinson disease: a phase 2 randomized clinical trial	JAMA Neurology	443
8	Mak M. K.	2017	Long-term effects of exercise and physical therapy in people with Parkinson disease	Nature Reviews Neurology	437
9	Hirsch M. A.	2003	The effects of balance training and high-intensity resistance training on persons with idiopathic Parkinson’s disease	Archives of Physical Medicine and Rehabilitation	400
10	Lim I.	2005	Effects of external rhythmical cueing on gait in patients with Parkinson’s disease: a systematic review	Clinical Rehabilitation	399
11	Fisher B. E.	2008	The effect of exercise training in improving motor performance and corticomotor excitability in people with early Parkinson’s disease	Archives of Physical Medicine and Rehabilitation	396
12	Ahlskog J. E.	2011	Does vigorous exercise have a neuroprotective effect in Parkinson disease?	Neurology	389
13	Keus S. H. J.	2007	Evidence-based analysis of physical therapy in Parkinson’s disease with recommendations for practice and research	Movement Disorders	385
14	Frenkel-Toledo S.	2005	Treadmill walking as an external pacemaker to improve gait rhythm and stability in Parkinson’s disease	Movement Disorders	356
15	Abbruzzese G.	2016	Rehabilitation for Parkinson’s disease: current outlook and future challenges	Parkinsonism and Related Disorders	352
16	Mirelman A.	2011	Virtual reality for gait training: can it induce motor learning to enhance complex walking and reduce fall risk in patients with Parkinson’s disease?	Journals of Gerontology: Series A, Biological Sciences and Medical Sciences	330
17	Kwok J. Y. Y.	2019	Effects of mindfulness yoga vs. stretching and resistance training exercises on anxiety and depression for people with Parkinson disease: a randomized clinical trial	JAMA Neurology	319

The remaining highly cited publications covered rhythmic auditory stimulation, remotely supervised aerobic exercise, high-intensity treadmill training, balance and resistance training, external cueing, physical therapy, exercise-related neuroplasticity, virtual-reality gait training, and mindfulness yoga. Collectively, these publications reflected the sustained importance of gait training, balance, aerobic exercise, physical therapy, motor learning, and neuroplasticity within the exercise-rehabilitation literature for people with Parkinson disease.

## 4 Discussion

This study mapped the development of research on exercise and exercise-based rehabilitation for people with Parkinson’s disease from the perspectives of publication trends, international collaboration, keyword co-occurrence, thematic evolution, outcome measures, and highly cited publications. The marked increase in publications after 2010 reflects more than a simple expansion in research volume. Gait, balance, rehabilitation, and motor function remained the stable core of the field across all four periods, whereas cognition, mood, quality of life, sleep, fatigue, digital assessment, remote intervention, and exercise dose progressively entered the main research network. This thematic evolution indicates a gradual deepening of the research agenda: early studies primarily examined whether exercise provided additional benefits beyond usual care; subsequent work focused on whether specific functional impairments could be improved through targeted training; and current research increasingly seeks to align intervention components and exercise dose with delivery settings, individual characteristics, and outcome selection.

### 4.1 Expansion of the research field has supported the development of an evidence base, but evidence quality and regional representativeness remain limited

The volume of publications increased rapidly after 2010, broadly coinciding with the transition of exercise from an adjunctive recommendation to a routine component of PD rehabilitation. However, the maturity of the evidence remains constrained by intervention heterogeneity and methodological limitations, particularly inconsistent classification of exercise modalities, inadequate reporting of exercise dose and progression rules, and substantial variation in outcome selection (Padilha et al., 2023). A Cochrane network meta-analysis of 154 randomized controlled trials reported a mean sample size of only 51 participants per trial, while only 85 trials provided safety information to any extent, indicating that small samples and incomplete safety reporting remain common (Ernst et al., 2024). Existing studies have also focused predominantly on short-term post-intervention changes, with relatively limited evaluation of benefit maintenance, long-term adherence, and transfer to daily-life functioning (Mak et al., 2017). A systematic review examining whether exercise can attenuate PD progression found only limited evidence and was unable to establish a consistent disease-modifying effect (Li et al., 2023). Current evidence therefore provides stronger support for short-term improvements in symptoms and function than for the superiority of a specific exercise modality, the long-term maintenance of benefits, or a stable disease-modifying effect. At present, the most defensible conclusion is that regular exercise is generally beneficial, rather than that a single optimal exercise approach has been identified for all people with PD and all outcomes.

Previous bibliometric studies have similarly shown that exercise research in PD has been concentrated in the United States and European countries, although research output from Asian countries, particularly China, has increased in recent years (Chen et al., 2022). However, absolute publication counts are influenced by national research capacity, database coverage, international collaboration, and counting methods and therefore cannot be used to compare research efficiency without appropriate standardization (Donthu et al., 2021). Accordingly, the country rankings in this study reflect only the absolute output captured by the selected databases and should not be interpreted as direct indicators of rehabilitation service quality, research quality, or population-adjusted research capacity. Much of the current PD research and clinical evidence also originates from high-income settings in Europe and North America, whereas low- and middle-income countries continue to face substantial limitations in research capacity, epidemiological data, and rehabilitation resources (Schiess et al., 2022). This geographic imbalance may restrict the external applicability of existing exercise-rehabilitation evidence across healthcare systems, cultural contexts, and resource settings.

Changes observed around 2020 should also be interpreted in the context of disruptions to rehabilitation services during the COVID-19 pandemic. Social restrictions and reduced access to face-to-face treatment made it more difficult for people with PD to maintain regular exercise while also accelerating the development of remotely delivered exercise interventions (Langer et al., 2021). A feasibility study conducted during the pandemic found that participants accompanied by caregivers attended more sessions, whereas pain, insufficient motor ability, reduced physical capacity, and fear of falling were major barriers to participation (Torriani-Pasin et al., 2022). Although participants generally recognized the health value and safety of remote exercise, most still preferred in-person training. Another implementation study of remote physical therapy similarly showed that both patients and therapists favored integrating telehealth with face-to-face assessment and treatment rather than replacing conventional rehabilitation entirely (Colón-Semenza et al., 2024). Remote rehabilitation should therefore be positioned as a complementary model for improving service accessibility and continuity, with its use tailored to individual risk, technological capacity, family support, and the need for in-person assessment.

### 4.2 The persistent centrality of gait and balance reflects a shift toward task-specific motor control in PD rehabilitation

Gait, balance, and motor function remained central throughout the study period, consistent with the high prevalence and disabling consequences of gait and postural-control impairments in PD and their direct effects on daily mobility and independent living (Bohnen et al., 2022). Difficulties with gait initiation, turning, freezing of gait, and falls cannot be explained solely by muscle weakness; they are also associated with impaired motor automaticity, abnormal postural control, disrupted sensory integration, and deficits in executive and attentional function (Peterson et al., 2016). Freezing of gait, in particular, has been associated with impaired response inhibition, attentional allocation, attentional switching, and visuospatial processing, indicating that complex walking depends on the interaction between cognitive and motor-control systems. Previous physical therapy research has also shown that training effects are often task- and context-specific and may not transfer spontaneously to untrained activities (Corrà et al., 2021).

Global motor rating scales provide useful measures of disease severity and overall motor burden, but a single scale cannot adequately capture the distinct constructs of posture, gait, balance, turning, and freezing of gait (Bloem et al., 2016). A systematic review of functional mobility measures in PD showed that the Timed Up and Go test, walking speed, walking endurance, and other task-based mobility measures assess different dimensions of function and should not be regarded as interchangeable (Bouça-Machado et al., 2020). As research objectives have shifted from overall symptom improvement toward actual mobility, outcome assessment should therefore extend beyond a single global scale to include straight-line walking, sit-to-stand and turning performance, dynamic balance, dual-task walking, and mobility in complex environments. Laboratory-based testing and daily-life monitoring capture different aspects of mobility, and their combined use may provide a more comprehensive evaluation of intervention effects. The RESCUE randomized controlled trial showed that 3 weeks of home-based rhythmic cueing produced small improvements in gait-related mobility, walking speed, step length, and freezing-of-gait severity, although some benefits diminished during post-intervention follow-up (Nieuwboer et al., 2007). These findings support the short-term clinical value of external rhythmic cueing for gait rehabilitation but do not establish that its effects can be maintained over the long term or consistently transferred to untrained contexts. The clinical practice guideline of the American Physical Therapy Association separately recommends external cueing, gait training, balance training, resistance training, and task-specific training and links these interventions to outcomes including freezing of gait, step length, walking speed, postural control, muscle strength, and functional mobility (Osborne et al., 2021). These recommendations indicate that PD exercise rehabilitation is moving away from general exercise advice toward task-specific interventions targeting defined functional impairments.

The value of different training approaches therefore lies not only in increasing physical activity but also in providing distinct sensory information, cognitive demands, and motor-learning conditions (Peterson et al., 2016). External cues can provide visual or auditory references for step length and gait rhythm and reduce reliance on impaired internally generated movement cues (Ginis et al., 2018). Motor-cognitive and dual-task training simultaneously impose walking and cognitive demands and target attentional allocation and motor coordination under dual-task conditions; meta-analytic evidence suggests that these interventions can improve dual-task performance, gait, and selected balance outcomes (Johansson et al., 2023). Virtual-reality training can incorporate obstacle negotiation, environmental variation, visual feedback, and decision-making into gait practice, thereby supporting complex walking and motor learning, although its additional benefits over conventional training require further confirmation (Mirelman et al., 2011). Progressive resistance training can improve lower-limb strength, but its effects on walking speed and balance are inconsistent, indicating that increased strength does not necessarily translate into improved gait or postural control (Tillman et al., 2015).

Improvements observed under training conditions may not transfer automatically to daily life. Reviews of cueing interventions for freezing of gait indicate that cueing may improve immediate post-training performance, whereas long-term consolidation and transfer to untrained situations remain limited (Ginis et al., 2018). Laboratory and home walking speeds show only moderate-to-high correlations and reflect different mobility behaviors; laboratory performance therefore cannot be assumed to represent daily-life mobility directly (Corrà et al., 2021). People with PD may also compensate for impaired step-length regulation by increasing cadence, meaning that a higher cadence accompanied by shorter steps does not independently demonstrate improved gait quality (Morris et al., 1994). Real-world digital gait studies also differ in devices, monitoring duration, definitions of walking bouts, analytical algorithms, and outcome reporting, limiting cross-study comparability and clinical interpretation (Kirk et al., 2025).

Based on this evidence, future research should select training content according to specific functional impairments rather than continue to compare broad exercise labels. Impaired rhythm and step-length regulation may be more appropriately addressed through external cueing; turning and mobility in complex environments require task-specific practice; muscle weakness requires progressive resistance training; and increased dual-task cost may require exercise that incorporates cognitive demands (Peterson et al., 2016). Prescribing exercise according to functional deficits and active training components is more consistent with a physical therapy framework based on continuous assessment and individualized adjustment of motor rehabilitation.

### 4.3 Expansion of multidimensional outcomes and digital technologies is driving PD exercise rehabilitation toward individualized prescription

Cognition, depression and anxiety, sleep, fatigue, and quality of life became increasingly prominent in recent years, indicating that outcome assessment in PD exercise research has expanded beyond motor symptoms toward multidimensional health outcomes. Large cohort studies have shown that both motor and neuropsychiatric symptoms are associated with health-related quality of life in people with PD, suggesting that motor function alone cannot fully represent the overall disease burden (Bock et al., 2022). Patient surveys have also identified fatigue, mental health, sleep, pain, and cognitive function as important priorities from the early stages of PD and across different disease durations (Port et al., 2021). Consequently, relying solely on motor ratings or walking tests may overlook non-motor symptoms and functional consequences that are important to patients when evaluating the overall clinical value of exercise interventions.

Cognition, mood, and quality of life should not, however, be automatically classified as patient-centered outcomes simply because they belong to non-motor domains. The importance assigned to symptoms and functional problems varies substantially among individuals and may change over the course of the disease; walking, balance, freezing of gait, fatigue, sleep, mood, and independence may all become priorities at different stages. Patient-centered assessment is defined by whether an outcome captures changes that patients consider important and meaningful, rather than by whether it belongs to a motor or non-motor category. Kang et al. demonstrated that individualized goal setting was feasible among people with PD and subjective cognitive decline and that rehabilitation goals covered a range of cognitive and daily-activity domains (Mammen et al., 2024). Cognition, mood, sleep, and quality of life are therefore more accurately described as non-motor or multidimensional health-related outcomes, whereas their patient-centered relevance should be established through individualized goals and patient-reported measures.

Current evidence suggests that exercise may improve selected non-motor outcomes, but the certainty of the evidence is inconsistent. A systematic review and meta-analysis by Folkerts et al. found that exercise may improve global cognition in people with PD, although the certainty of evidence was very low and the most effective exercise type could not be identified (Folkerts et al., 2024). A randomized controlled trial by Amara et al. showed that 16 weeks of high-intensity resistance and interval training improved sleep efficiency, total sleep time, wake after sleep onset, and slow-wave sleep; however, these findings were derived from a specific intensity and training protocol and cannot be generalized to all exercise modalities (Amara et al., 2020). Longitudinal cohort evidence has also linked worsening sleep quality with increasing fatigue and depression, indicating that these non-motor outcomes are interrelated and that changes in a single outcome may not be attributable entirely to exercise (Koh et al., 2022). The increasing frequency of these themes therefore primarily reflects growing research attention and should not be interpreted as evidence that treatment effects or underlying mechanisms have been conclusively established.

Different exercise modalities may influence multidimensional outcomes through different active components. Motor-cognitive training, dual-task training, and virtual reality commonly incorporate attentional allocation, task switching, spatial processing, and feedback-based learning, whereas Tai Chi, Qigong, and yoga combine postural control, rhythm, breathing, and attentional regulation. Their effects may arise from the combined influence of physical loading, cognitive engagement, and social interaction rather than from a single mechanism (Jin et al., 2020; Johansson et al., 2023; Marotta et al., 2022). Future studies should use component-controlled comparisons, factorial trials, or mediation analyses to distinguish the independent and interactive effects of specific intervention components.

Exercise dose is central to the transition from comparing exercise modalities to developing evidence-based prescriptions. Shulman et al. found that low-intensity treadmill training improved six-minute walking distance to a similar or greater extent than high-intensity treadmill training, whereas stretching and resistance training produced greater improvements in muscle strength, indicating that higher intensity does not necessarily yield superior effects across all outcomes (Shulman et al., 2013). The Park-in-Shape trial showed that people with mild PD could complete 6 months of remotely supervised home-based aerobic exercise and achieve improvements in selected motor outcomes, although its long-term effects and potential disease-modifying properties require further investigation (Kolk et al., 2019). These findings indicate that exercise dose should be interpreted in relation to the target outcome and intended population rather than treating intensity alone as an indicator of treatment superiority.

Individualized prescription requires more than specifying an exercise modality. Frequency, intensity, session duration, intervention length, progression, supervision, and the dose actually completed should all be reported. Disease stage, fall risk, cognitive capacity, frailty, multimorbidity, and family support may influence both exercise tolerance and treatment response. Rather than searching for a universally optimal exercise modality, identifying safe, sustainable, and clinically meaningful training combinations for specific functional profiles and risk levels is more consistent with the practical requirements of PD rehabilitation (Mcginley and Nakayama, 2024). Digital technologies provide new opportunities for assessment and prescription adjustment. The recent growth of technology-assisted rehabilitation may also have coincided with the broader commercial availability and declining cost of head-mounted displays and remote-monitoring technologies, although the present analysis did not formally test specific market milestones. However, most existing studies have focused on correlations between sensor-derived variables and clinical measures, whereas direct evidence that these technologies improve clinical decision-making or health outcomes remains limited (Moreau et al., 2023; Sapienza et al., 2024). Digital technologies have therefore increased measurement frequency and environmental coverage but have not yet consistently translated into improved treatment quality.

Remote rehabilitation can reduce transportation and geographic barriers and support the continuity of home-based training, but its suitability is influenced by cognitive ability, technological competence, safety risk, family support, and cost. Implementation studies suggest that remote delivery is generally feasible, although study participants are often selectively recruited and some assessments and interventions still require face-to-face contact (Colón-Semenza et al., 2024; Laar et al., 2023). A more practical model may therefore integrate in-person assessment, professional supervision, home-based exercise, and continuous monitoring rather than replacing conventional rehabilitation entirely with remote care.

Overall, current evidence supports the development of explicit links among functional phenotype, active training components, exercise dose, delivery setting, and target outcomes. Freezing of gait, balance impairment, muscle weakness, and dual-task difficulties arise from different functional mechanisms and should not be addressed using identical training content or outcome measures. Bibliometric analysis can describe research themes, collaboration structures, and knowledge evolution but cannot directly determine treatment effects, risk of bias, or certainty of evidence. The emerging topics identified in this study should therefore be regarded as priorities for further clinical investigation rather than established therapeutic conclusions.

## 5 Conclusion

This bibliometric and visualization analysis mapped 6,542 English-language original articles and reviews on exercise and exercise-based rehabilitation for people with PD indexed in the Web of Science Core Collection and Scopus through June 15, 2026. Publication activity accelerated after 2010 and developed into an internationally connected field, with substantial contributions from North America and Europe and rapidly increasing output from Asia.

Gait, rehabilitation, balance, motor function, and quality of life remained the stable thematic core, while cognition, depression and anxiety, sleep, fatigue, digital rehabilitation, and exercise-dose optimization became increasingly prominent. The evolution of the literature reflects a transition from basic functional rehabilitation toward multidimensional assessment, technology-assisted delivery, and more individualized exercise management.

Future studies should standardize reporting of intervention components and exercise dose, harmonize motor and non-motor outcome measurement, extend follow-up into real-world settings, improve representation across regions and patient subgroups, and directly test the mechanisms and modifiers of exercise response. Bibliometric findings describe the structure and direction of the research field but do not establish treatment effectiveness or certainty of evidence; clinical decisions should continue to rely on rigorously conducted trials, systematic reviews, and clinical practice guidelines.

## 6 Limitations

The literature sample was limited to the Web of Science Core Collection and Scopus. These databases were selected for broad coverage and standardized citation metadata, but they do not index every relevant clinical, rehabilitation, nursing, allied-health, or regional publication. CINAHL, PubMed/MEDLINE, Embase, the Cochrane Library, SPORTDiscus, PEDro, and non-English regional databases were not searched. The findings should therefore be interpreted as a mapping of literature indexed in the two selected databases rather than an exhaustive census of all exercise research involving people with PD. Country and continental results were based on absolute counts and were not normalized by population size, research workforce, or research expenditure. Article type was separated in the temporal analysis but was not stratified by country, which limits interpretation of whether national output consisted primarily of original studies or reviews.

Cross-database differences in author names, institutional affiliations, journal titles, keywords, document types, and citation formats may have produced residual duplicates or incomplete merging despite normalization procedures. Broad interdisciplinary reviews could enter the dataset when their indexed titles, abstracts, or keywords contained both PD- and exercise-related terms; consequently, global citation rankings may favor highly cited broad-scope publications that are not specific exercise trials. Citation counts indicate visibility rather than relevance, methodological quality, or evidentiary strength. The retracted publication in Table 1 was retained only to describe the retrieved citation record and was excluded from substantive interpretation.

Keyword networks, burst detection, thematic maps, period definitions, and forecasts depend on the search strategy, cleaning thesaurus, software algorithms, and parameter settings. The four periods were selected from inflection points in publication growth, but alternative cut points could yield different thematic structures. Forecasts were exploratory, and 2026 represented an incomplete year. The analysis did not formally test the effects of the COVID-19 pandemic or commercial technology milestones on publication trends. Finally, bibliometric methods do not directly assess risk of bias, treatment effects, safety, or certainty of evidence; these questions require complementary appraisal of trials, systematic reviews, meta-analyses, and clinical guidelines.
